# Case Report: PD-1 Inhibitor Is Active in Lung Adenocarcinoma With B Cell Deficiency

**DOI:** 10.3389/fimmu.2020.563622

**Published:** 2020-11-06

**Authors:** Shumin Yuan, Xiufeng Hu, Yanqiu Zhao, Zibing Wang

**Affiliations:** ^1^ Department of Immunotherapy, Affiliated Cancer Hospital of Zhengzhou University & Henan Cancer Hospital, Zhengzhou, China; ^2^ Department of Internal Medicine, Affiliated Cancer Hospital of Zhengzhou University & Henan Cancer Hospital, Zhengzhou, China

**Keywords:** immunotherapy, immune checkpoint inhibitor, PD-1, non-small cell lung cancer, B cells

## Abstract

**Introduction:**

In murine cancer models, B cells are unnecessary for efficacy of PD-1 inhibitor. However, we do not know whether this applies to clinical settings, especially in patients with non-small-cell lung carcinoma (NSCLC).

**Case presentation:**

We report on the case of an advanced lung adenocarcinoma patient without oncogenic driver mutations whose disease progressed on second-line bevacizumab-containing chemotherapy regimens. These previous treatments resulted in profound thrombocytopenia and increased number of B cells; both effects were hard to alleviate. The patient was diagnosed with marginal zone B-cell lymphoma by flow cytometry immunophenotyping. After five cycles of rituximab in combination with lenalidomide treatment, the percentage of B cells rapidly declined to undetectable levels and the lymphoma regressed completely. However, because masses in the lung gradually increased, this patient was subsequently treated with a PD-1 inhibitor. The patient’s condition stabilized, and the mass shrank to reach partial response, with progression free survival exceeding 15 months and no serious adverse events.

**Conclusion:**

The present case proves the efficacy of PD-1 inhibitor in metastatic lung adenocarcinoma in the absence of B cells. Immune checkpoint inhibitions are thus a choice for patients with B cell deficiencies, such as X-linked agammaglobulinemia, immunoglobulin deficiencies, and common variable immunodeficiency, diseases that have historically been excluded from clinical trials for oncologic drugs.

## Introduction

Clinical trial and real-world data have revealed that PD-1 inhibitor monotherapy is effective in NSCLC patients as second-line treatment and beyond, but only for a small proportion of patients (1). Various factors limit the efficacy of PD-1 inhibitor in the majority patients, but among them, the formation of immunosuppressive tumor microenvironment is critical (2). Since B cells inhibit induction of T cell-dependent tumor immunity (3), B cells might be one factor affecting the efficacy of PD-1 inhibitor. Support for this comes from a recent study indicating that PD-1 inhibitor still exerts activity when B cells are depleted using anti-CD20 mAbs and in B cell-deficient mice using tumor-bearing mouse models (4). However, we do not know whether this is also the case in a clinical setting, especially for patients with NSCLC.

Here, we describe a unique case of a metastatic NSCLC patient treated with rituximab to deplete B cells, followed by PD-1 inhibitor. We discuss the efficacy and adverse effects of PD-1 inhibitor in the absence of B cells.

## Case Presentation

On October 20^th^, 2017, a 67-year-old man was hospitalized because of wheezing and chest tightness. His personal history included 40-years of smoking 20 cigarettes per day. No notable medical history was present.

During admission as an inpatient to hospital, chest computed tomography (CT) showed a 37 × 25 mm soft tissue mass shadow in the lower lobe of the left lung, an 11 × 8 mm nodule in the lower lobe of the right lung, and lymph node metastases in the right supraclavicular region, 2L and 7 regions of mediastinum, and left hilum. No abnormalities were found in the head and bone. A biopsy of a supraclavicular lymph node was performed, and its findings confirmed metastasis of lung adenocarcinoma without EGFR, ALK, ROS1, RET, or MET mutations.

**Figure 1:**
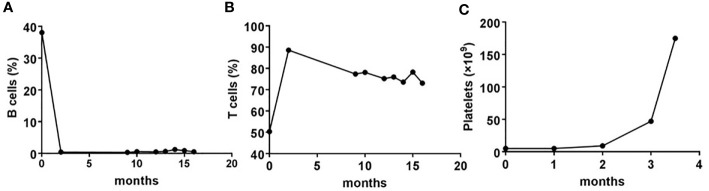
The dynamic changes in number of B, T, and platelet pre- and post-rituximab in combination with lenalidomide treatment. **(A, B)** Whole blood cells, obtained from the patient before and after each cycle of treatment with rituximab plus lenalidomide, were stained with CD19 or CD3 monoclonal antibodies, and analyzed using flow cytometry. Shown are frequencies, which were expressed as a percentage of nuclear cells. **(C)** Routine blood examinations were performed before and after each cycle of treatment with rituximab plus lenalidomide. Shown are the number of platelets, which were expressed as cells/L.

In November 2017, the patient was enrolled into one clinical trial receiving the bevacizumab in combination with paclitaxel and carboplatin regimen for four cycles. CT scans showed a partial response of the disease. Tolerance, however, was poor, with grade III thrombocytopenia. Subsequently, the regimen switched to pemetrexed in combination with bevacizumab for three cycles. Although a CT scan revealed a further partial response after these cycles of treatments, progressive thrombocytopenia occurred and reached the lowest point on June 20^th^, 2018 (platelet count, 3 × 10^9^/L). After extensive discussion at the multidisciplinary team (MDT) meeting, a bone marrow aspiration was done to examine the reason for thrombocytopenia. The results of the bone marrow smears showed that the ratio of granulocyte and erythroid series was 22.5:1, granulocytes accounted for 25%–50% of the bone marrow cells, with non-hematopoietic cells predominant, and the proportion of mature lymphocytes was 73.2%. Flow cytometry analysis of the bone marrow aspirate showed an abnormal mature B cell lymphoid subset, accounting for 30.3% of nuclear cells and expressing CD19, HLA-DR, CD20, FMC7, CD79b, and surface immunoglobulin Kappa. Marginal zone lymphoma was diagnosed. This patient received 5 cycles of rituximab in combination with lenalidomide treatment. At the end of the treatment, lymphoma was in complete remission. The platelet number resumed to normal level, the B cell number decreased to zero level, while the T cell number was unaffected (**Figure 1**).

**Figure 2:**
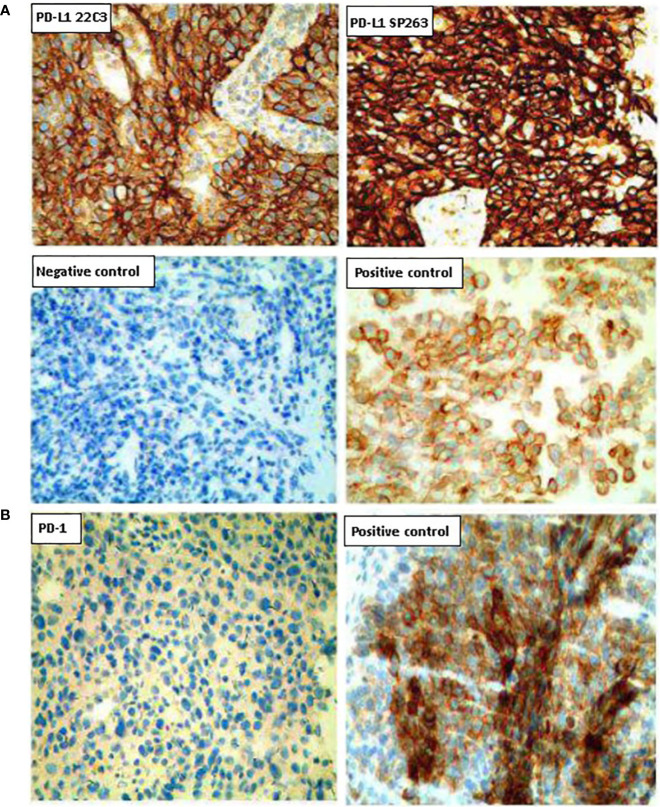
Representative images of PD-L1 and PD-1 expression on tumor tissues. **(A)** Tumor sections were stained with the PD-L1 IHC 22C3 pharmDx (Agilent) and the IVD VENTANA PD-L1 (SP263). **(B)** Tumor sections were stained with the PD-1 antibody. Magnification (200×).

In February 2019, a CT scan revealed disease progression, with the mass in the lower lobe of the left lung approximately 52 × 41 mm. Bone scans showed that bone metabolism was active in the right clavicle, the ninth and twelfth thoracic vertebra, and the first and second lumbar vertebrae, indicating bone metastasis (**Supplemental Figure 1**). A second biopsy was performed on the lung mass and adenocarcinoma was confirmed. The analysis of gene mutations showed that TP53 gene was mutated and MET gene was amplified. Results from PD-L1 immunohistochemical staining showed that the rate of PD-L1 positive tumor cells was 90%, and that of PD-1 positive cells less than 1% (**Figure 2**).

**Figure 3:**
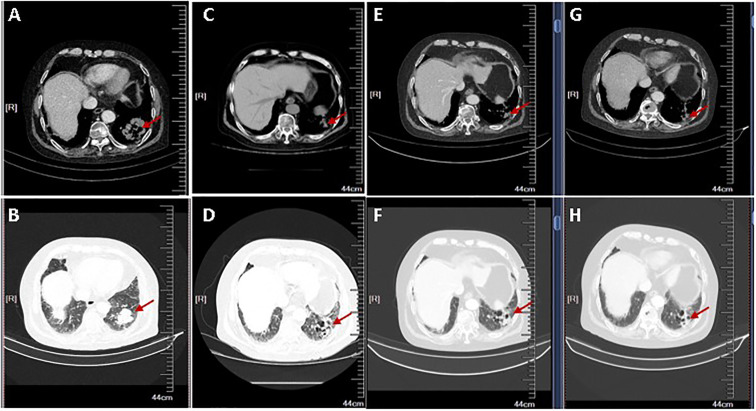
The radiological tumor evolution. Shown are two different views of chest computed tomography (CT) slices (**A, C, E, G**: Chest window; **B, D, F, H**: Lung window). **(A, B)** CT scan showing mass of the lung adenocarcinoma pre-treatment of PD-1 inhibitor. **(C–H)** CT scan shows a partial response after 2 **(C, D)**, 4 **(E, F)**, and 6 **(G, H)** cycles of immunotherapy. The arrow shows the changes of the left lung mass during the PD-1 administration.

**Figure 4:**
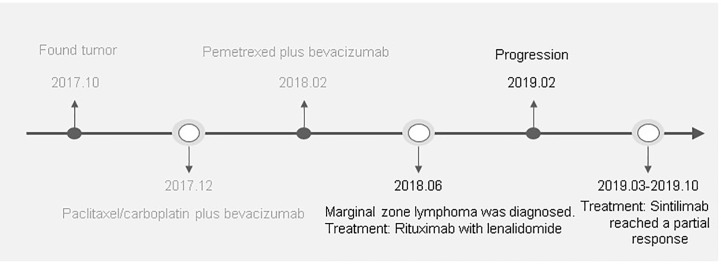
A detailed clinical timeline is displayed. On October 20, 2017, the patient was hospitalized. After a series of examinations, the patient was diagnosed with advanced lung adenocarcinoma without driver gene mutations. In December, 2017, the patient was started on a chemotherapy regimen containing paclitaxel/carboplatin plus bevacizumab for 4 cycles. Due to grade III thrombocytopenia, the regimen was subsequently switched to pemetrexed plus bevacizumab for 3 cycles from February, 2018. Although a CT scan revealed a partial response after these cycles of treatments, progressive thrombocytopenia occurred. After extensive examination, the patient was diagnosed marginal zone lymphoma in June, 2018. After 5 cycles of rituximab in combination with lenalidomide treatment, lymphoma was in complete remission. However, a CT in February, 2019 demonstrated disease progression. From March, 2019 to October, 2019 the patient was treated with sintilimab, a PD-1 inhibitor, of 200 mg every 3 weeks. CT imaging demonstrated significant treatment responses.

Given that PD-L1 were highly expressed on tumor cells, the MDT decided to treat the patient with sintilimab injection, a PD-1 inhibitor, of 200 mg every 3 weeks, starting in March 2019. After four cycles, a CT scan revealed a 38.4% reduction in the soft tissue mass shadow in the lower lobe of the left lung to about 27 × 32 mm, reaching a partial response (**Figure 3**). Because the patient refused to undergo bone scans, we do not know whether the patient’s suspected bone metastases have also been relieved. However, from the results of each CT examination, there was no significant increase in bone metastasis, and the patient did not have corresponding pain symptoms. The patient continued PD-1 inhibitor monotherapy every 3 weeks, and no adverse events have occurred. In October 2019, after 10 treatments and 8 months of disease control, the patient’s condition was stable and the lung mass had shrunk further. At the time of writing, treatment with immunotherapy is ongoing (**Figure 4**).

## Discussion

We describe the case of a metastatic NSCLC patient whose B cells were depleted using rituximab, but who still responded well to PD-1 inhibitor with no adverse effect. The present case reveals several points.

B cells are unnecessary for efficacy of PD-1 inhibitor in patients with NSCLC. PD-1 inhibitor monotherapy has shown clinical effectiveness for the treatment of patients with advanced NSCLC, leading to approval of PD-1 as a second-line treatment for such patients. However, it only confers an objective response rate of 20% and a 5-year survival rate of 16% (5). The low efficacy for most patients results from multiple mechanisms. A suppressive tumor microenvironment enriched for inhibitory cells poses a major obstacle for cancer immunity (6). Besides regulatory T cells and myeloid-derived suppressor cells, B cells are a limiting factor for T cell-mediated antitumor immunity in earlier studies. In addition, we have preliminary data that high levels of B cells influence the overall objective response rate of PD-1 inhibitor (Frontiers in Immunology, in press). We analyzed baseline B cell levels in the peripheral blood of 79 patients with advanced malignant solid tumors following treatment with PD-1 inhibitor-based anti-tumor approaches and assessed its association to clinical efficacy. Patients with partial responses exhibited lower levels of B cells compared to those with progressive disease (Frontiers in Immunology, in press). These observations suggest that B cells reduce the efficacy of PD-1 inhibitor and that elimination of B cells does not significantly affect anti-tumor immunity induced by PD-1 inhibitor. This idea is bolstered by a recent mouse study showing that anti-PD-1 responses were no different in the absence of B cells using murine colon cancer and melanoma models (4), and through the present case, showing that depletion of B cells using rituximab had no effect on NSCLC response to a PD-1 inhibitor.

The use of a PD-1 inhibitor plus treatments that remove B cells may be used for the subgroup of NSCLC patients that have preexisting autoimmune diseases. B cells, autoantibodies, and T cells are all important components of abnormal immune responses that lead to tissue pathology unique to each autoimmune disease. For systemic lupus erythematosus, rheumatoid arthritis, scleroderma, type 1 diabetes, and multiple sclerosis, B cells are regarded as key mediators of the autoimmune disease and are closely correlated with disease activity (7). For immune checkpoint inhibitor related autoimmune diseases, B cells are an important contributor to autoimmunity following immune checkpoint inhibitor use (8). Rituximab, originally approved to treat B-cell lymphoma, is an anti-CD20 monoclonal antibody that targets B cells but is increasingly used for the treatment of many autoimmune conditions such as rheumatoid arthritis, granulomatosis with polyangiitis, and other antineutrophil cytoplasmic antibody-associated vasulitides (9). Thus, rituximab can be combined with PD-1 inhibitor to treat patients with NSCLC and concurrent autoimmune diseases, without affecting clinical efficacy of NSCLC to PD-1 inhibitor and with potential clinical efficacy of autoimmune diseases to rituximab.

PD-1 inhibitor plus treatments to remove B cells may be used for patients with NSCLC and concurrent B-cell lymphoma. One clinical trial has shown that PD-1 inhibitor has antitumor activity in relapsed/refractory primary mediastinal large B-cell lymphoma patients treated with rituximab (10) and a number of ongoing clinical trials are assessing the activity of PD-1 inhibitor in combination with rituximab in B cell lymphomas. Yet, it remains unknown whether PD-1 inhibitor are effective in patients with NSCLC and concurrent B cell lymphoma post-rituximab treatment. Although the two diseases did not occur simultaneously at diagnosis, the present case gave us the information that B cell depletion using rituximab does not affect the efficacy of PD-1 inhibitor in patients with NSCLC.

The frequency and severity of adverse effects related to PD-1 inhibitor might be decreased when B cells are depleted in NSCLC patients. NSCLC patients receiving PD-1 inhibitor often have adverse effects, including autoimmune hypophysitis, thyroiditis, colitis, hepatitis, pneumonitis, and rash, sometimes appearing as systemic diseases (11). Thus, patients with NSCLC and concurrent autoimmune disease have typically been excluded from clinical trials using PD-1 inhibitor due to their underlying immune disorders. However, from a practical perspective, it would be highly desirable to identify whether patients with concurrent autoimmune diseases can be treated with a PD-1 inhibitor, whether autoimmunity is aggravated if PD-1 inhibitor is administrated, and whether autoimmunity is reduced by preemptive intervention, without affecting clinical efficacy. In a real-world multi-institutional retrospective study, although the use of a PD-1 inhibitor in patients with NSCLC and a history of autoimmune disease led to 22% overall response rate, it led to exacerbation of the autoimmune disease in 23% of patients (12). Changes in B cell subsets strongly correlate with the risk of immune-related adverse events (8). These observations provide the basis for exploration of B cell-targeted therapies as a preemptive approach to improve tolerance to PD-1 inhibitor for at-risk individuals. The present case showed no adverse effects, suggesting that the frequency and severity of adverse effects related to PD-1 inhibitor might be decreased when B cells are depleted in NSCLC patients.

## Concluding Remarks

Taken together, the present case suggests that PD-1 inhibitor is effective and safe in the absence of B cells, and that a PD-1 inhibitor plus rituximab might be used for NSCLC patients that concurrently suffer from an autoimmune disorder or lymphoma. This deserves to be further investigated using large prospective clinical trials.

## Data Availability Statement

The raw data supporting the conclusions of this article will be made available by the authors, without undue reservation.

## Ethics Statement

Written informed consent was obtained from the individual(s) for the publication of any potentially identifiable images or data included in this article.

## Author Contributions

SY wrote the paper. XH and YZ provided technical or material support. ZW has designed the paper. All authors contributed to the article and approved the submitted version.

## Funding

This work was supported by the National Natural Science Foundation of China (Grant No. 81972690, 81000914, and 81272526).

## Conflict of Interest

The authors declare that the research was conducted in the absence of any commercial or financial relationships that could be construed as a potential conflict of interest.
