# Supplementary figures and images for: Case Report: PD-1 Inhibitor Is Active in Lung Adenocarcinoma With B Cell Deficiency
Source: Front Immunol. 2020 Nov 6;11:563622. doi: 10.3389/fimmu.2020.563622 (PMC7681247; doi:10.3389/fimmu.2020.563622)

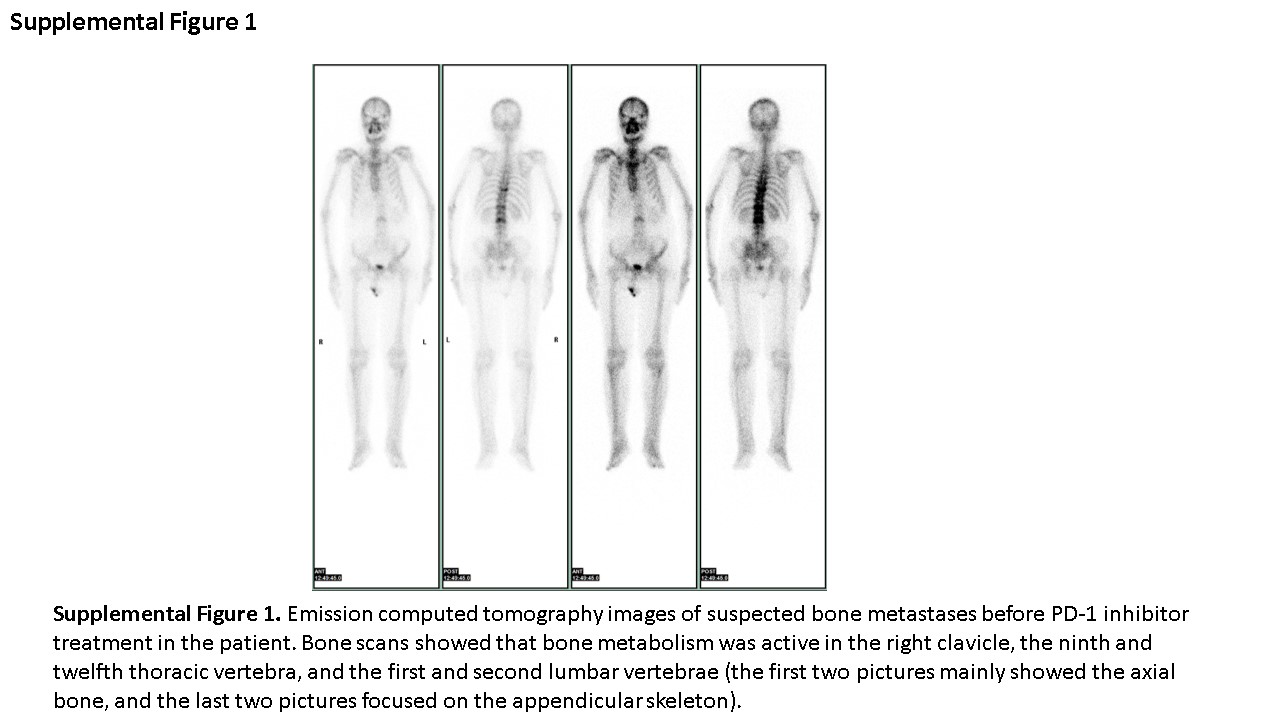

Supplement: Supplementary file 1 [file Image_1.jpeg]
